# Creating a switchable optical cavity with controllable quantum-state mapping between two modes

**DOI:** 10.1038/s41598-018-32989-9

**Published:** 2018-10-03

**Authors:** Grzegorz Chimczak, Karol Bartkiewicz, Zbigniew Ficek, Ryszard Tanaś

**Affiliations:** 1 Faculty of Physics, Adam Mickiewicz University, PL-61-614 Poznań, Poland; 2 RCPTM, Joint Laboratory of Optics of Palacký University and Institute of Physics of Academy of Sciences of the Czech Republic, 17. listopadu 12, 772 07 Olomouc, Czech Republic; 3 National Centre for Nanotechnology and Advanced Materials, KACST, P.O. Box 6086, Riyadh, 11442 Saudi Arabia; 4 Quantum Optics and Engineering Division, Institute of Physics, University of Zielona Góra, Szafrana 4a, Zielona Góra, 65-516 Poland

## Abstract

We describe how an ensemble of four-level atoms in the diamond-type configuration can be applied to create a fully controllable effective coupling between two cavity modes. The diamond-type configuration allows one to use a bimodal cavity that supports modes of different frequencies or different circular polarisations, because each mode is coupled only to its own transition. This system can be used for mapping a quantum state of one cavity mode onto the other mode on demand. Additionally, it can serve as a fast opening high-Q cavity system that can be easily and coherently controlled with laser fields.

## Introduction

Quantum systems, in which a control of the coherent evolution is possible, are of great importance from a theoretical and a practical points of view, and therefore, such systems always attract research interest^1,2^. Systems composed of a cavity and atoms, which are trapped inside this cavity, are such systems because one can easily control the evolution of their quantum state just by illuminating atoms with a laser^3–7^. Moreover atom-cavity systems provide a versatile environment for engineering complex non-classical states of light^8–17^. Researchers achieve such high level of control over the evolution of quantum states employing atoms, which can be modelled by few special level schemes. The simplest and frequently considered schemes are three level atoms in Λ and *V* configurations^18–25^. The main advantage of these atoms is the possibility of working with the two-photon Raman transition involving an intermediate level, which is populated only virtually during the whole evolution. Since atoms are driven by a classical laser field, the Raman transition takes place only if the laser is turned on. The same idea allows for full control of the system evolution in many other level schemes. Therefore researchers have used and studied intensively many different types of atoms coupled to the cavity mode^26–36^. There is, however, one important atomic level scheme, which is almost ignored by researchers in the context of atom-cavity systems–a four-level atom in the diamond configuration (a ⬦-type atom, also known as a double-ladder four-level atom). Despite the fact that this level scheme is rich in quantum interference and coherence features^37^ and has many other applications^38–45^, to the best of our knowledge there are only few articles about the ⬦-type atom coupled to the quantized field modes^46–57^.

**Figure 1:**
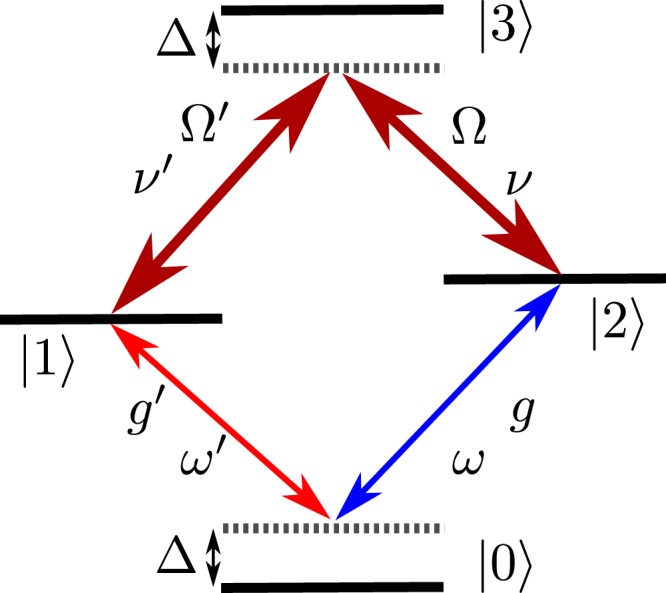
Energy levels of an atom in the diamond configuration. Lower atomic transitions are coupled to quantized field modes with frequencies *ω* and *ω*′. Upper transitions are driven by classical laser fields with frequencies *ν* and *ν*′.

In this paper we study a ⬦-type atom interacting with two quantized cavity modes and two classical laser fields. The quantized field modes are coupled to lower atomic transitions while the classical laser fields are coupled to upper atomic transitions, as depicted in Fig. 1. Here, we show that under certain conditions this system creates effective coupling between two cavity modes of different frequencies or different circular polarisations and its evolution can be described by a simple effective Hamiltonian, and can be easily controlled just by switching the lasers on and off. Moreover, we show that it is possible to increase the effective coupling strength between both modes by using an ensemble of four-level ⬦-type atoms, which interact collectively with both modes. We also present two applications of this system. First of them is a mapping of an arbitrary state of light from one mode onto the other. By *mapping* we mean an operation, which is defined by the transformation equation ${({\sum }_{k}{c}_{k}|k\rangle }_{A})\otimes \mathrm{|0}{\rangle }_{B}\to \mathrm{|0}{\rangle }_{A}\otimes {({\sum }_{k}{c}_{k}|k\rangle }_{B})$^58^. This operation makes it possible to transfer coherent superpositions of cavity-mode number states from the mode labeled by *A* to the mode labeled by *B*. Second application is a device that plays the role of an effective cavity, in which we can change the effective Q factor on demand just by turning the lasers on and off. This device is based on the scheme proposed by Tufarelli *et al*.^59^ but it employs ⬦-type atoms instead of two-level atoms which makes the physics of the described system much richer. Thus, the proposed system has the potential to be more versatile and efficient in quantum information processing than the solutions based on the two-level atoms.

This paper is organized as follows. Firstly, we present the effective Hamiltonian of simple form that governs the evolution of the analyzed complex quantum system and we study conditions under which the effective Hamiltonian can be considered as a good approximation of the general Hamiltonian. Secondly, we investigate the behaviour of the system in two different cases, when lasers are turned off and in the limit of high-intensity laser fields, to show that we can control the effective coupling between two cavity modes just by switching the lasers on and off. Thirdly, we prove using the effective Hamiltonian that the system can perform a state-mapping operation between these two modes. Fourthly, we present the basic idea of the second application of the system, *i.e*., a fast opening high-Q cavity. Lastly, we discuss in detail the system performance for this second application.

## Results

### Effective description of the system

We consider an ensemble of *n* identical four-level atoms in the diamond configuration (Fig. 1) with a ground level |0〉, two non-degenerate intermediate levels |1〉, |2〉, and an upper level |3〉. There are four allowed transitions in this level scheme. The |0〉 ↔ |2〉 transition is coupled to the field mode represented by the annihilation operator *a* with coupling strength *g*, while the |0〉 ↔ |1〉 transition is coupled to the field mode described by the annihilation operator *b* with coupling strength *g* ′. The frequency of the *a* mode is *ω* and the frequency of the *b* mode is *ω*′. Both field modes are equally detuned from the corresponding transition frequencies by ${\rm{\Delta }}=({E}_{1}-{E}_{0})/\hslash -\omega ^{\prime} =({E}_{2}-{E}_{0})/\hslash -\omega$. The upper transitions |1〉 ↔ |3〉 and |2〉 ↔ |3〉 are driven by coherent laser fields of frequencies *ν*′ and *ν*, respectively. The coupling strengths between these atomic transitions and the laser fields are denoted by Ω′ and Ω. Both laser fields are detuned from the corresponding transition frequencies by Δ. Simultaneously, the atom is coupled to all other modes of the EM field, which are assumed to be in the vacuum state. The atom provides an effective coupling between both the modes. Of course, the effective coupling strength depends on the number of atoms *n*. The higher the number of atoms *n*, the stronger the coupling becomes. We assume that there are *n* ≥ 1 identical ⬦-type four-level atoms trapped inside the cavity. The evolution of this composite quantum system is governed by the Hamiltonian, which in the rotating frame is given by

1 $$\begin{array}{rcl}H & = & \sum _{k=1}^{n}\{{\rm{\Delta }}{\sigma }_{11}^{(k)}+{\rm{\Delta }}{\sigma }_{22}^{(k)}+2{\rm{\Delta }}{\sigma }_{33}^{(k)}\\  &  & +({\rm{\Omega }}{\sigma }_{23}^{(k)}+{\rm{\Omega }}^{\prime} {\sigma }_{13}^{(k)}+g{a}^{\dagger }{\sigma }_{02}^{(k)}+g^{\prime} {b}^{\dagger }{\sigma }_{01}^{(k)}+{\rm{h}}{\rm{.c}}{\rm{.}})\},\end{array}$$

where $\hslash =1$ and ${\sigma }_{ij}^{(k)}=|i{\rangle }_{k}\langle j|$ denotes the atomic flip operator between states |*i*〉_*k*_ and |*j*〉_*k*_ for the *k* th atom. The Lindblad operators representing spontaneous transitions from the atomic excited states are given by

2 $${L}_{1}^{(k)}=\sqrt{\gamma ^{\prime} }{\sigma }_{01}^{(k)},\,{L}_{2}^{(k)}=\sqrt{\gamma }{\sigma }_{02}^{(k)},\,{L}_{3}^{(k)}=\sqrt{{\gamma }_{3}}{\sigma }_{23}^{(k)},\,{L}_{4}^{(k)}=\sqrt{{\gamma ^{\prime} }_{3}}{\sigma }_{13}^{(k)},$$

where *γ*, *γ*′, *γ*_3_ and *γ*′_3_ are spontaneous emission rates for the respective transitions. For the sake of simplicity, we assume that Ω, Ω′, *g* and *g* ′ are real and non-negative. Similar four-level scheme has been proposed in ref.^28^. The diamond configuration, however, has the advantage that it allows to use (contrary to level scheme of ref.^28^) atomic transitions with the highest values of the dipole moment. Of course, the higher the dipole moment, the stronger the effective coupling between the modes is. Using the method of adiabatic elimination (see Methods) we derive the following effective Hamiltonian

3 $${H}_{{\rm{eff}}}={\delta }_{0}({a}^{\dagger }a+{b}^{\dagger }b)+{\delta }_{1}{b}^{\dagger }b+{\delta }_{2}({a}^{\dagger }b+{b}^{\dagger }a),$$

where *δ*_0_ = −*ng*^2^*α*_2_, *δ*_1_ = *n*(*g*^2^*α*_2_ − *g*′^2^*α*_1_) and *δ*_2_ = −*ngg*′*α*_3_, for *α*_1_ = *ξ*(Ω^2^ − 2Δ^2^), *α*_2_ = *ξ*(Ω′^2^ − 2Δ^2^), *α*_3_ = −*ξ*ΩΩ′, *α*_4_ = −*ξ*Δ^2^, *α*_5_ = *ξ*ΔΩ′, *α*_6_ = *ξ*ΔΩ with *ξ* = 1/(Δ[Ω^2^ + Ω′^2^ − 2Δ^2^]). This effective Hamiltonian (3) works properly if populations of all atomic excited states are small (see Alexanian-Bose method in Methods). The effective master equation

4 $$\dot{\rho }=-\,i[{H}_{{\rm{eff}}},\rho ]+\sum _{j=1}^{2}\{{L}_{{\rm{eff}}}^{(j)}\rho {({L}_{{\rm{eff}}}^{(j)})}^{\dagger }-\,\frac{1}{2}[{({L}_{{\rm{eff}}}^{(j)})}^{\dagger }{L}_{{\rm{eff}}}^{(j)}\rho +\rho {({L}_{{\rm{eff}}}^{(j)})}^{\dagger }{L}_{{\rm{eff}}}^{(j)}]\},$$

where

5 $$\begin{array}{rcl}{L}_{{\rm{eff}}}^{\mathrm{(1)}} & = & \sqrt{n\gamma ^{\prime} }[{\alpha }_{3}\,ga+{\alpha }_{1}\,g^{\prime} b],\\ {L}_{{\rm{eff}}}^{\mathrm{(2)}} & = & \sqrt{n\gamma }[{\alpha }_{2}\,ga+{\alpha }_{3}\,g^{\prime} b],\end{array}$$

requires more restrictive conditions to work properly, because in its derivation (see Reiter-Sørensen method in Methods) we have neglected the Lindblad operators ${L}_{3}^{(k)}$ and ${L}_{4}^{(k)}$, which describe spontaneous emissions from the upper states |3〉_*k*_. Therefore, we assume that populations of the atomic intermediate levels (|1〉_*k*_ and |2〉_*k*_) are small and populations of the upper states |3〉_*k*_ are small even compared with the intermediate levels, because then probabilities of occurrence of collapses described by ${L}_{3}^{(k)}$ and ${L}_{4}^{(k)}$ are negligibly small. It is necessary to know conditions for the parameters, which make these assumptions true. We restrict ourselves only to cases where Ω′*g* ≈ Ω*g*′. In these cases the effective master equation works properly if the following conditions are satisfied

6 $$|{\rm{\Delta }}|\gg {g}_{{\rm{\min }}}\sqrt{n}\,{\rm{\max }}(\sqrt{\langle {a}^{\dagger }a\rangle },\sqrt{\langle {b}^{\dagger }b\rangle })\,{\rm{and}}\,{\rm{\max }}({\lambda }_{2},{\lambda }_{3},{\lambda }_{5},{\lambda }_{6})\ll \,{\rm{\min }}({\lambda }_{1},{\lambda }_{4}),$$

where *g*_min_ = min(*g*, *g* ′) and *λ*_*i*_ are dimensionless expansion parameters (see Alexanian-Bose method in Methods for the definition of *λ*_*i*_ parameters). The expansion parameters *λ*_2_, *λ*_3_, *λ*_5_ and *λ*_6_ are associated with operators acting on the states |3〉_*k*_. The smaller they are, the smaller are the populations of the states |3〉_*k*_. The expansion parameters *λ*_1_ and *λ*_4_ are associated with operators acting only on the states |1〉_*k*_ and |2〉_*k*_. Knowing values of *g* and *g*′ of the chosen physical system we set the value of Δ according to the first condition and then we find numerically the value of Ω for which the second condition is satisfied. We can always find such value of Ω, because when intensities of classical fields tend to infinity, then expansion parameters *λ*_2_, *λ*_3_, *λ*_5_, *λ*_6_ tend to zero. In the following text we are going to use the effective master eq. (4).

Let us consider the dynamics of the four-level atom in the diamond configuration in the limit of high-intensity classical fields. In the dressed-state approach there is one ground atomic state |0〉 and three excited states

7 $$\begin{array}{rcl}|\mu \rangle  & = & {{\mathscr{N}}}_{\mu }(\,-\,{\rm{\Omega }}\mathrm{|1}\rangle +{\rm{\Omega }}^{\prime} \mathrm{|2}\rangle ),\\ |\varphi \rangle  & = & {{\mathscr{N}}}_{\varphi }\mathrm{(2}{\rm{\Omega }}^{\prime} \mathrm{|1}\rangle +2{\rm{\Omega }}\mathrm{|2}\rangle +({\rm{\Delta }}-{{\rm{\Omega }}}_{{\rm{R}}})\mathrm{|3}\rangle ),\\ |\psi \rangle  & = & {{\mathscr{N}}}_{\psi }(2{\rm{\Omega }}^{\prime} \mathrm{|1}\rangle +2{\rm{\Omega }}\mathrm{|2}\rangle +({\rm{\Delta }}+{{\rm{\Omega }}}_{{\rm{R}}})\mathrm{|3}\rangle ),\end{array}$$

where ${{\mathscr{N}}}_{\mu },{{\mathscr{N}}}_{\varphi }\,\mathrm{and}\,{{\mathscr{N}}}_{\psi }$ are normalisation factors and Ω_R_ = (Δ^2^ + 4Ω^2^ + 4Ω′^2^)^1/2^. Here, there are three allowed transitions: |0〉 ↔ |*μ*〉, |0〉 ↔ |*ϕ*〉 and |0〉 ↔ |*ψ*〉, each of which is coupled to both cavity modes (see Alexian-Bose method in Methods). As mentioned above, when intensities of classical fields tend to infinity, then expansion parameters *λ*_2_, *λ*_3_, *λ*_5_, *λ*_6_ tend to zero. It means that only two atomic levels, *i.e*. |0〉 and |*μ*〉, are enough to describe the evolution of the system–the four-level atom in the diamond configuration effectively works exactly in the same way as the detuned two-level atom in this regime. Note that the excited bare state |3〉 can be then neglected. It might seem counter-intuitive that high coupling strengths between the atomic upper transitions and the laser fields lead to an effective decoupling of the upper level |3〉 from the system dynamics, but this idea is known and discussed for example in ref.^60^.

In the limit of high-intensity classical fields one more thing is clearly seen from the first condition in (6)—the effective coupling strength *δ*_2_ scales as $\sqrt{n}$. Such behaviour is the well known feature of the collective dynamics^61–63^.

When the lasers are turned off then the evolution of the system is still governed by the Hamiltonian (1) but with Ω = Ω′ = 0. The formulas for the effective Hamiltonian given by eq. (3) and the effective operators in this case read as

8 $${H}_{{\rm{eff}}}=-\,(n{g}^{2}/{\rm{\Delta }}){a}^{\dagger }a-(ng{^{\prime} }^{2}/{\rm{\Delta }}){b}^{\dagger }b,\,{L}_{{\rm{eff}}}^{\mathrm{(1)}}=\sqrt{n\gamma ^{\prime} }(g^{\prime} /{\rm{\Delta }})b,\,{L}_{{\rm{eff}}}^{\mathrm{(2)}}=\sqrt{n\gamma }(g/{\rm{\Delta }})a.$$

Here, we can also easily derive more precise expressions, if we perform the adiabatic elimination of excited atomic states assuming from the start that Ω = Ω′ = 0. This approach results in

9 $${H}_{{\rm{eff}}}=-\,\frac{n{g}^{2}{\rm{\Delta }}}{{{\rm{\Delta }}}^{2}+{\gamma }^{2}/4}{a}^{\dagger }a-\,\frac{ng{^{\prime} }^{2}{\rm{\Delta }}}{{{\rm{\Delta }}}^{2}+\gamma {^{\prime} }^{2}/4}{b}^{\dagger }b,\,{L}_{{\rm{eff}}}^{\mathrm{(1)}}=\frac{\sqrt{n\gamma ^{\prime} }g^{\prime} }{{\rm{\Delta }}-i\gamma ^{\prime} \mathrm{/2}}b,\,{L}_{{\rm{eff}}}^{\mathrm{(2)}}=\frac{\sqrt{n\gamma }g}{{\rm{\Delta }}-i\gamma /2}a.$$

It is important to note that there is no coupling between the two cavity modes, and therefore, there is no photon transfer when the lasers are turned off. We will refer to this working mode of the system as to *the closed mode*.

### Quantum-state mapping between two cavity modes

Under certain conditions the evolution of a complex system formed by an ensemble of four-level diamond-type atoms interacting with two quantized field modes can be easily controlled just by switching the lasers on and off. Let us now demonstrate that we can use this system to transfer a given quantum state of one mode (for example a qudit or the Schrödinger′s cat states) to the other mode on demand. It has shown that in special cases, *i.e*., for coherent states and for qubit states, the Hamiltonian of the form (3) can swap the states of the two modes^26,27^. Here, we show that it is possible to transfer an arbitrary photonic state.

First, we need the formula for the average photon number in the mode represented by the annihilation operator *b*, assuming that initially this mode is empty, while the mode represented by *a* is prepared in the Fock state |*n*_ph_〉. This formula will help us investigate the photon transfer process. We can derive it introducing the superposition bosonic operator of both field modes

10 $$C=\sqrt{1-\varepsilon \,}a-\sqrt{\varepsilon \,}b.$$

We choose such *ε* that the Hamiltonian (3) can be expressed in the form ${H}_{{\rm{eff}}}=-\,{\delta }_{r}{C}^{\dagger }C$, where ${\delta }_{r}=$ ${(4{\delta }_{2}^{2}+{\delta }_{1}^{2})}^{\mathrm{1/2}}$. Using this form of the Hamiltonian one can derive the formula for the average photon number

11 $$\langle {b}^{\dagger }b\rangle ={n}_{{\rm{ph}}}(1-{\delta }_{1}^{2}/{\delta }_{r}^{2}){\sin }^{2}({\delta }_{r}t/2).$$

**Figure 2:**
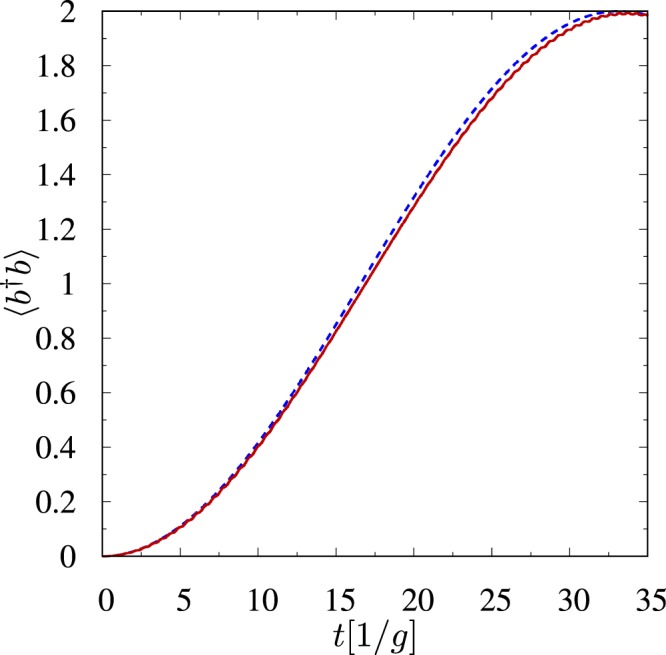
The average photon number in the *b* mode as a function of time calculated numerically (solid line) and given by eq. (11) (dashed line) for one atom *n* = 1 and (*g*′, Δ, Ω, Ω′, *γ*, *γ*′, *γ*′′)/*g* = (1, 11, 55, 55, 1, 1, 1), where *g*/2*π* = 10 MHz. At *t* = 0 the *a* mode is prepared in the state |2〉_*A*_, while the *b* mode is in a vacuum state. After the *π* pulse both photons are transferred to the *b* mode.

In Fig. 2 we plot the average photon number as a function of time. This figure shows that all photons can be transferred from the first mode to the second mode. However, this is possible only if *δ*_1_ = 0. We want the state mapping to be perfect, and therefore, we restrict ourselves to this case only. We can make *δ*_1_ ≈ 0 by choosing values of Ω and Ω′, which are much greater than Δ and satisfy condition Ω′*g* ≈ Ω*g*′. If one wants *δ*_1_ = 0 then values of Ω and Ω′ have to be chosen more precisely

12 $${\rm{\Omega }}^{\prime} =\sqrt{({{\rm{\Omega }}}^{2}-2{{\rm{\Delta }}}^{2})g{^{\prime} }^{2}/{g}^{2}+2{{\rm{\Delta }}}^{2}}.$$

For reference, we also calculate numerically the average photon number using the non-Hermitian Hamiltonian

13 $$\begin{array}{rcl}\tilde{H} & = & ({\rm{\Delta }}-i\gamma ^{\prime} /2){\sigma }_{11}+({\rm{\Delta }}-i\gamma /2){\sigma }_{22}+(2{\rm{\Delta }}-i\gamma ^{\prime\prime} /2){\sigma }_{33}\\  &  & +({\rm{\Omega }}{\sigma }_{23}+{\rm{\Omega }}^{\prime} {\sigma }_{13}+g{a}^{\dagger }{\sigma }_{02}+g^{\prime} {b}^{\dagger }{\sigma }_{01}+{\rm{h}}{\rm{.c}}{\rm{.}}),\end{array}$$

which governs the evolution of this open system during the time intervals when no collapse occurs^64,65^. We have obtained the Hamiltonian (13) by substituting the relevant symbols in

14 $$\tilde{H}=H-\,\frac{i}{2}\sum _{j}{L}_{j}^{\dagger }{L}_{j}$$

with quantities from eqs (1) and (2) for *n* = 1. As one can see from Fig. 2, the analytical results are in a remarkable agreement with the numerical solution even for quite considerable values of *γ*, *γ*′ and *γ*′′ (where $\gamma ^{\prime\prime} ={\gamma }_{3}+{\gamma ^{\prime} }_{3}$) as long as parameter regime justifies adiabatic elimination.

From eq. (11) we can infer that the *π* pulse time is given by the formula

15 $${t}_{\pi }=\pi /{\delta }_{r},$$

from which one can observe one more important feature of the Hamiltonian. It is evident that the time of such *π* pulse is independent of *n*_ph_, and thus, we are able to perform the state-mapping operation defined by |*n*_ph_〉_*A*_ ⊗ |0〉_*B*_ → |0〉_*A*_ ⊗ |*n*_ph_〉_*B*_. Let us move into the rotating frame, in which the Hamiltonian takes the form

16 $${H}_{{\rm{eff}}}=-\,{\delta }_{2}({a}^{\dagger }a+{b}^{\dagger }b)+{\delta }_{2}({a}^{\dagger }b+{b}^{\dagger }a)\,$$

and let us assume that the first mode is initially prepared in some interesting quantum state $|{{\rm{\Psi }}}_{0}\rangle ={\sum }_{k}{c}_{k}|k{\rangle }_{A}$, while the second mode is empty. Then, by switching the lasers on for *t*_*π*_, one can map this interesting state onto the second mode

17 $$(\sum _{k}{c}_{k}|k{\rangle }_{A})\otimes \mathrm{|0}{\rangle }_{B}\to \mathrm{|0}{\rangle }_{A}\otimes (\sum _{k}{c}_{k}|k{\rangle }_{B}).$$

**Figure 3:**
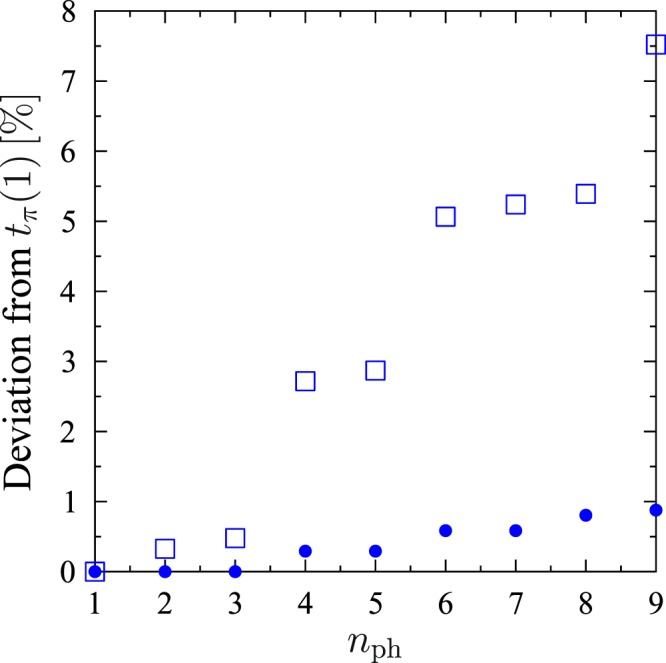
Deviation of *t*_*π*_(*n*_ph_) from *t*_*π*_(1) (in percent) for (*g*′, Δ, Ω, Ω′)/*g* = (1, 10, 33, 33) (open squares) and for (*g*′, Δ, Ω, Ω′)/*g* = (1, 30, 100, 100) (solid circles). The second parameter regime justifies adiabatic elimination for *n*_ph_ = 9, whereas the first one only for *n*_ph_ = 1.

In a frame rotating at different frequency, in which the Hamiltonian takes the form

18 $${H}_{{\rm{eff}}}={\delta }_{x}({a}^{\dagger }a+{b}^{\dagger }b)+{\delta }_{2}({a}^{\dagger }b+{b}^{\dagger }a),$$

phase factors appear and the *π* pulse changes the initial state according to

19 $$(\sum _{k}{c}_{k}|k{\rangle }_{A})\otimes \mathrm{|0}{\rangle }_{B}\to \mathrm{|0}{\rangle }_{A}\otimes (\sum _{k}{c}_{k}{e}^{i{\varphi }_{\pi }(k)}|k{\rangle }_{B}),$$

where *ϕ*_*π*_(*n*_ph_) = −*n*_ph_*π*(*δ*_2_ + *δ*_*x*_)/(2*δ*_2_). Note that for the parameters values used in Fig. 2 *δ*_0_ = −2.74 and *δ*_2_ = 2.98, so *δ*_0_ ≈ −*δ*_2_. For the Hamiltonian (3), *δ*_1_ = 0 and large Ω there are no phase factors, because *δ*_0_ tends to −*δ*_2_ for large Ω, and thus, the Hamiltonian (3) tends to the form given by eq. (16). The independence of *t*_*π*_ from *n*_ph_ is crucial for the state-mapping operation. Unfortunately, *t*_*π*_ is independent of *n*_ph_ only in the approximated model (3), in which we adiabatically eliminated all atomic excited levels. Numerical calculations show that *t*_*π*_ increases with *n*_ph_ in the more general model of the system given by the Hamiltonian (1) for *n* = 1. However, as long as the adiabatic elimination is justified, we can neglect the dependence *t*_*π*_ on *n*_ph_, as is seen in Fig. 3. It is seen from Fig. 3 that there are jumps of the value of *t*_*π*_. These jumps come from the fact that populations of atomic excited levels oscillate with high frequencies^66–68^. Thus, there are many local closely-spaced maxima of the population of the desired final state |0〉_*A*_ ⊗ |*n*_ph_〉_*B*_. Therefore, the global maximum (*t*_*π*_) changes sometimes discontinuously with increasing of *n*_ph_–from one local minimum to the next one. We can neglect these jumps as long as the adiabatic elimination is justified.

Let us now investigate the effect of *γ* and *γ*′ on the state-mapping operation. To this end we need non-Hermitian Hamiltonian, which we obtain by inserting Eq. (3) and the relevant effective rotating frame Lindblad operators (see Reiter-Sørensen method in Methods) into eq. (14). Assuming that ${\rm{\Omega }},{\rm{\Omega }}^{\prime} \gg {\rm{\Delta }}$ and *δ*_1_ = 0, this Hamiltonian can be quite well approximated by

20 $$\tilde{H}=-\,2{\delta }_{2}{C}^{\dagger }C-\frac{i}{2}{\gamma }_{{\rm{eff}}}{C}^{\dagger }C,$$

where the effective dissipation rate is given by

21 $${\gamma }_{{\rm{eff}}}=\frac{2n{g}^{2}g{^{\prime} }^{2}({g}^{2}\gamma ^{\prime} +g{^{\prime} }^{2}\gamma )}{{{\rm{\Delta }}}^{2}{({g}^{2}+g{^{\prime} }^{2})}^{2}}.$$

It is clear that the fidelity of the state mapping ${\mathcal F}$ and the probability that no collapse occurs during this operation ${\mathscr{P}}$ are close to one only if the effective dissipation rate *γ*_eff_ is much less than the effective coupling strength *δ*_2_. For *g* = *g*′ and *γ* = *γ*′, the expression for the effective dissipation rate takes the simpler form ${\gamma }_{{\rm{e}}{\rm{f}}{\rm{f}}}=n\gamma {g}^{2}/{{\rm{\Delta }}}^{2}$. In this special case, ${\mathcal F}$ and ${\mathscr{P}}$ depend on the ratio *γ*/Δ. Let us now check this result numerically using the non-Hermitian Hamiltonian

22 $$\begin{array}{rcl}\tilde{H} & = & \sum _{k=1}^{n}\{({\rm{\Delta }}-i\gamma ^{\prime} \mathrm{/2}){\sigma }_{11}^{(k)}+({\rm{\Delta }}-i\gamma \mathrm{/2}){\sigma }_{22}^{(k)}+(2{\rm{\Delta }}-i\gamma ^{\prime\prime} /2){\sigma }_{33}^{(k)}\\  &  & +({\rm{\Omega }}{\sigma }_{23}^{(k)}+{\rm{\Omega }}^{\prime} {\sigma }_{13}^{(k)}+g{a}^{\dagger }{\sigma }_{02}^{(k)}+g^{\prime} {b}^{\dagger }{\sigma }_{01}^{(k)}+{\rm{h}}{\rm{.c}}{\rm{.}})\}.\end{array}$$

First, we have to choose specific values of parameters. The choice of the atom-cavity system determines *g*, *g*′, *γ*, *γ*′ and *γ*′′. For macroscopic cavities *g*/2*π* is typically of the order of 10 MHz and *γ* ranges from about 0.2 *g* to *g*^4,36^. Let us set *g*′ = *g* = 2*π*⋅10 MHz, *γ*′ = *γ* = 2 *g* and *γ*′′ = *g*. The choice of the initial state determines the Fock state |*n*_ph_〉, to which the state mapping has to be faithful. Let the initial state of the *a* mode be |Ψ_0_〉 = (|0〉_*A*_ + |1〉_*A*_ + |2〉_*A*_ + |3〉_*A*_)/2. If there are four atoms trapped in the cavity, then the detuning has to satisfy ${\rm{\Delta }}\gg g\sqrt{4\cdot 3}$. We set Δ = 35 *g*. Finally, we choose the value of Ω and calculate Ω′ using eq. (12). These values have to be large enough to satisfy the second condition in (6). It is easy to check that for Ω = Ω′ = 175 *g* this condition is fulfilled, and therefore, adiabatic elimination is justified. For (*g*′, Δ, Ω, Ω′, *γ*, *γ*′, *γ*′′)/*g* = (1, 35, 175, 175, 2, 2, 1) and *n* = 4 we have found that ${\mathcal F} =0.993$ and ${\mathscr{P}}=0.885$. In the case of one atom trapped in the cavity (*n* = 1), for the same parameters, we have found that ${\mathcal F} =0.995$ and ${\mathscr{P}}=0.886$. One can see that ${\mathcal F}$ and ${\mathscr{P}}$ are almost the same in the two cases. The only important difference is the time of the state-mapping operation–*t*_*π*_ = 26.5/*g* and *t*_*π*_ = 105.6/*g* for *n* = 4 and *n* = 1, respectively. The time of the state mapping in the one-atom case is almost four times larger than that in the four-atom case. This result is in an agreement with eq. (15). We can make *t*_*π*_ smaller in the one-atom case by setting smaller Δ but then the ratio *γ*/Δ increases and the dissipation reduces the fidelity and the success probability. For instance, if we set (*g*′, Δ, Ω, Ω′, *γ*, *γ*′, *γ*′′)/*g* = (1, 17, 85, 85, 2, 2, 1) in the one-atom case then the time of the state mapping is reduced to *t*_*π*_ = 51.6/*g*. Then, however the dissipation reduces the fidelity and the success probability to ${\mathcal F} =0.979$ and ${\mathscr{P}}=0.795$, respectively.

### Quantum-state extraction by fast opening high-Q cavity

The investigated system can be applied as a fast opening high-Q cavity that can be easily and coherently controlled with classical laser fields. The device is based on similar principles as the setup of Tufarelli *et al*.^59^, but it employs four-level atoms in the diamond configuration instead of two-level atoms. The main idea of both setups is to couple a high-Q cavity mode to a low-Q cavity mode through atoms. Such a device would be very useful, because on the one hand we need a high Q factor to reach the strong coupling regime^4,6,69–74^, in which we can generate a complex non-classical state of light trapped inside optical resonator^8–17^. On the other hand, we need a low Q factor to extract this state from the resonator into a waveguide before it will be distorted by the cavity damping. The device proposed by Tufarelli *et al*.^59^ makes it possible to change the effective Q factor. If atoms are absent, there is no coupling between the two modes and the whole system works as an effective high-Q cavity. If we move atoms into the cavity, then photons leak out of the high-Q mode through the low-Q mode and the whole device works as an effective low-Q cavity. Instead of shifting the atoms out of the cavity we can shift atoms out of resonance using a laser and the dynamic Stark effect. As long as the laser illuminates atoms, there is no coupling between modes. Here, we propose to replace two-level atoms by four-level atoms in the diamond configuration. Our modification allows us to use a bimodal cavity, which supports circularly polarised modes of the same or different polarisations and frequencies. Moreover, it requires intense laser light to illuminate atoms only in short time intervals, when we need the coupling between modes. When the laser is switched off, there is no coupling between modes.

## Discussion

**Figure 4:**
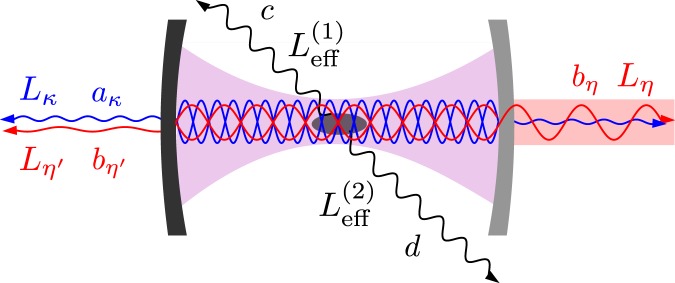
The effective model of the setup and the waveguide. The photon emission from the open cavity into the waveguide is represented by the operator *L*_*η*_. All photon losses are modelled by four inaccessible travelling modes. Emissions to these modes are described by *L*_*η*′_, *L*_*κ*_, ${L}_{{\rm{eff}}}^{\mathrm{(1)}}$ and ${L}_{{\rm{eff}}}^{\mathrm{(2)}}$.

After the adiabatic elimination of atomic excited states we can restrict our considerations to a simplified model, which does not include atomic variables. Such simplified model makes it easy to take into account all photon losses. To this end, we model the device as two cavity modes, which decay emitting the radiation into five travelling modes, as is depicted in Fig. 4. One of these travelling modes is accessible experimentally. This accessible travelling mode can be, for example, a waveguide. Other travelling modes are inaccessible, and thus, provide losses. The photon emissions from both cavity modes (represented by operators *a* and *b*) into the inaccessible travelling modes are described by the Lindblad operators: ${L}_{\eta ^{\prime} }=\sqrt{\eta ^{\prime} }b$, ${L}_{\kappa }=\sqrt{\kappa }a$, ${L}_{{\rm{eff}}}^{\mathrm{(1)}}$ and ${L}_{{\rm{eff}}}^{\mathrm{(2)}}$. The photon emission into the accessible travelling mode is described by the Lindblad operator ${L}_{\eta }=\sqrt{\eta }b$. Here we assume, unless explicitly stated otherwise, that the device is working in *the open mode*, *i.e*., both lasers are turned on (Ω, Ω′ ≠ 0). We also assume that the quantum state of field was prepared in advance in the mode represented by the operator *a*. Under these assumptions, we derived a quantity that describes the quality of the field extracted from the resonator into a waveguide. We refer to this quantity as to the figure of merit of the proposed device (see Methods). Let us now investigate the usefulness of the considered device to extract a field state from the *a* mode. We assume that there is only one optical cavity. This cavity supports two electromagnetic field modes of different frequencies *ω* and *ω*′ (see Fig. 4). The first of them is considered as the *a* mode, while the second one as the *b* mode. Each cavity mirror is described by its radius of curvature *r*, transmission coefficients *T* and *T*′ for the *a* mode and the *b* mode, respectively, and loss coefficient *L*, which is assumed to be the same for both modes. The *a* mode requires very low values of *T* and *L* for both mirrors. To our knowledge, these parameters take the lowest value for the mirror that has been used in the experiment of refs^36,75^. We set these values in our calculations, *i.e*., *T*_small_ = *T*_1_ = *T*_2_ = *T*′_1_ = 1.8 ppm and *L* = 3.15 ppm, where the subscripts indicate the mirror. The radius of curvature of both mirrors is 50 mm^36,75^. Now we can vary only the cavity length *l* and the transmission coefficient *T*′_2_, and therefore, we want to plot the figure of merit *F* as a function of these two quantities. First, we have to choose a concrete realisation of the ⬦-type atom. This kind of atomic level scheme has been investigated experimentally using different atoms. The ⬦-type configuration has been realised using cesium^40^ and rubidium atoms^41–44^. From refs^76,77^ we can infer that it is also possible to obtain the ⬦-type configuration using sodium dimers. We can also choose different levels, which can form the diamond configuration. For example in rubidium atoms we can choose levels (5*S*_1/2_, 5*P*_1/2_, 5*P*_3/2_, 6*S*_1/2_)^41^, (5*S*_1/2_, 5*P*_1/2_, 5*P*_3/2_, 5*D*_3/2_)^42–44^ or (5*S*_1/2_, 5*P*_3/2_, 6*P*_3/2_, 5*D*_5/2_)^56^. Here, we propose to use a ^87^Rb atom and its levels |5*S*_1/2_, *F* = 2, *m*_*F*_ = 2〉, |5*P*_3/2_,*F* = 3,*m*_*F*_ = 3〉, |6*P*_3/2_, *F* = 3, *m*_*F*_ = 3〉 and |6*D*_3/2_, *F* = 3, *m*_*F*_ = 3〉 to serve as |0〉, |1〉, |2〉 and |3〉, respectively. This choice determines values of modes frequencies to be *ω*/2*π* = 713.28 THz and *ω*′/2*π* = 384.23 THz^78^. The lifetimes of all used here excited levels can be found in ref.^79^. It is important that the lifetime of the level |3〉 is longer (*τ*_3_ = 256 ns) than lifetimes of the other excited levels (*τ*_1_ = 112 ns for |1〉 and *τ*_2_ = 26.25 ns for |2〉). So our assumption that spontaneous emissions from the excited level |3〉 can be neglected in calculations is justified not only by small population of this level, but also by ${\tau }_{3} > {\tau }_{1}$ and ${\tau }_{3}\gg {\tau }_{2}$. The spontaneous emissions can take the ^87^Rb atom from the states |1〉 and |2〉 only to state |0〉. Hence, it is easy to calculate corresponding spontaneous emission rates: *γ*/2*π* = 1.42 MHz and *γ*′/2*π* = 6.06 MHz. In principle, the scheme presented here works properly even with only one trapped atom. In real experiments, however, this scheme will require a much larger number of atoms to achieve the figure of merit that is close to unity. In order to compare our scheme with the original scheme of Tufarelli *et al*.^59^, we set here the same number of atoms as in ref.^59^, *i.e*., *n* = 1000. Trapping 1000 rubidium atoms and preparing them in the |5*S*_1/2_, *F* = 2, *m*_*F*_ = 2〉 state is possible using fiber-based Fabry-Perot cavities^80,81^. We have chosen the macroscopic cavity in our considerations. A number of atoms trapped inside macroscopic cavities is typically of the order of 10^5^ ^82^. Trapping ~1000 atoms also should be possible. Now we can calculate the coupling strength *g* using

23 $$g=\sqrt{\frac{3\pi {c}^{3}\gamma }{2{\omega }^{2}V}},$$

where *c* is the speed of light and *V* is the cavity mode volume given by

24 $$V=\pi cl\sqrt{l(2r-l)}/(4\omega ).$$

In order to calculate the coupling strength *g*′ we have to replace *ω* and *γ* by *ω*′ and *γ*′ in (23) and (24). The cavity damping constants of the considered scheme can be calculated as

25 $$\kappa =c(1-R)/(l\sqrt{R}),\,\eta =T{^{\prime} }_{2}{\eta }_{{\rm{tot}}}/{\mathscr{N}},\,\eta ^{\prime} =(2L+T{^{\prime} }_{1}){\eta }_{{\rm{tot}}}/{\mathscr{N}},$$

where *R* = 1 − *L* − *T*_small_, $${\mathscr{N}}=2L+T{^{\prime} }_{1}+T{^{\prime} }_{2}$$ and

26 $${\eta }_{{\rm{tot}}}=c\frac{1-\sqrt{R(1-L-T{^{\prime} }_{2})}}{l{R}^{\mathrm{1/4}}{(1-L-T{^{\prime} }_{2})}^{\mathrm{1/4}}}.$$

Finally, we have to fix values of Δ, Ω and Ω′. It is necessary to choose these values carefully. On the one hand, they should be big enough to make adiabatic elimination justified. On the other hand, they cannot be too big, because *δ*_2_ and extraction efficiency decrease with increasing Δ. In our computations we set Δ/*g* = 700 and Ω/Δ = 5, which justifies adiabatic elimination for cavity states with $\langle {a}^{\dagger }a\rangle \lesssim 10$. Then Ω′ is given by (12).

**Figure 5:**
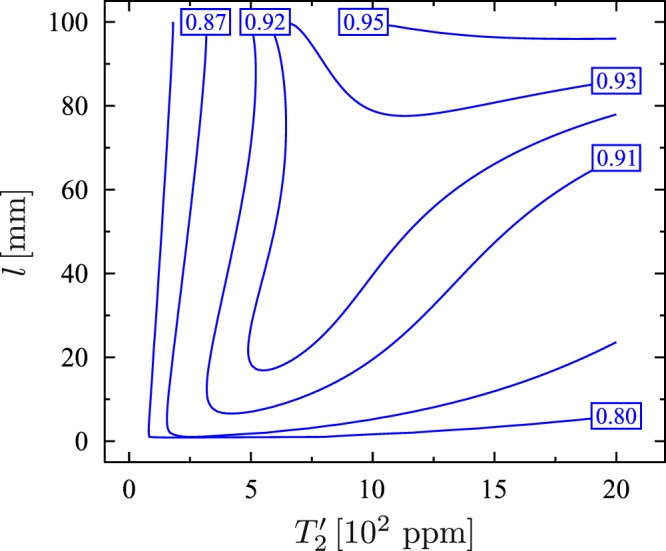
Figure of merit *F* as a function of the cavity length *l* and the transmission coefficient *T*′_2_ for 1000 ⬦-type four-level atoms.

**Figure 6:**
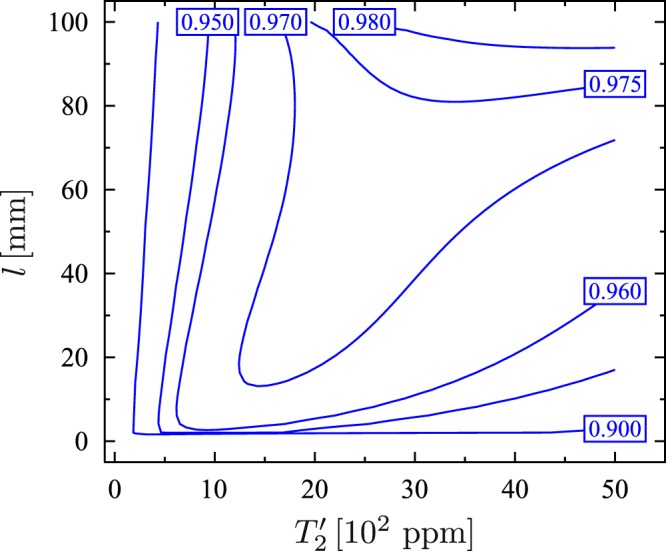
Figure of merit *F* as a function of the cavity length *l* and the transmission coefficient *T*′_2_ for 8000 ⬦-type four-level atoms.

Now, we can discuss the experimental feasibility of the scheme. In order to do so let us use figure of merit *F* closely related to the probability of successful operation of the discussed device. Under certain conditions (given explicitly in Methods) *F* is given as

27 $$F=\frac{\eta }{{\eta }_{{\rm{tot}}}}[1-\frac{{(\sqrt{{\zeta }_{1}{\theta }_{1}}+\sqrt{{\zeta }_{2}{\theta }_{2}})}^{2}}{2{\delta }_{2}^{2}}-\,\frac{{\eta }_{{\rm{tot}}}(\kappa +{\zeta }_{1}+{\zeta }_{2})}{4{\delta }_{2}^{2}+{\eta }_{{\rm{tot}}}(\kappa +{\zeta }_{1}+{\zeta }_{2})}],$$

where ${\zeta }_{1}=n\gamma ^{\prime} {\alpha }_{3}^{2}{g}^{2}$, ${\theta }_{1}=n\gamma ^{\prime} {\alpha }_{1}^{2}g{^{\prime} }^{2}$, ${\zeta }_{2}=n\gamma {\alpha }_{2}^{2}{g}^{2}$, ${\theta }_{2}=n\gamma {\alpha }_{3}^{2}g{^{\prime} }^{2}$, *η*_tot_ = *η*′ + *η*. We can plot *F* as a function of *l* and ${T^{\prime} }_{2}$. For the parameters given above the formula (27) gives only raw approximation of the figure of merit. Therefore, we have calculated *F* numerically using its definition (given in Methods), and we have obtained in this way results presented in Fig. 5. As expected, the figure of merit takes the maximum value in the near-concentric regime *l* ≈ 2*r*. For *l* = 99.9 mm and ${T^{\prime} }_{2}$ = 2000 ppm the figure of merit is equal to 0.97. Unfortunately, the near-concentric configuration of the macroscopic mirrors is extremely sensitive to misalignment, and therefore, it would be difficult or even impossible to achieve such high value of *F*^83^. For the confocal configuration *l* = *r*, which is the most stable configuration, the figure of merit can be equal to 0.92. This value is still quite high and it is higher than *F* of the original scheme of Tufarelli *et al*.^59^. Of course, we can always increase the figure of merit by increasing *n*. To show this we plot also the figure of merit for *n* = 8000. It is seen from Fig. 6 that now *F* = 0.97 even for the confocal configuration. Form eq. (27) it follows that the figure of merit can be close to one only under the condition $4{\delta }_{2}^{2}\gg {\eta }_{{\rm{tot}}}(\kappa +{\zeta }_{1}+{\zeta }_{2})$. Assuming *δ*_1_ = 0, this condition can be expressed as:

28 $${\eta }_{{\rm{tot}}}\ll n{g}^{2}/\gamma ,{\eta }_{{\rm{tot}}}\ll ng{^{\prime} }^{2}/\gamma ^{\prime} ,{\eta }_{{\rm{tot}}}\ll {\delta }_{2}({\delta }_{2}/\kappa ).$$

It follows that *κ* has to be at least two orders of magnitude smaller than *δ*_2_. For currently available atom-cavity systems all these conditions can be satisfied only for a large number of atoms *n*. Note that *F* is independent of *δ*_1_ in the mentioned regime. Typically, *g* ≠ *g*′ in concrete realisations of the four-level atom, and therefore, usually *δ*_1_ ≠ 0. A non-zero value of *δ*_1_ decreases *F* when dissipative rates are too large. It is possible to make *δ*_1_ = 0 by setting appropriate value of Ω′, *i.e*., this one given by eq. (12). From eq. (27), it is seen that such precise setting of Ω′ is not necessary in the regime, in which the figure of merit is close to one. This feature makes choosing values of parameters easier. It is also worth to note that in the regime ${\rm{\Omega }}\gg {\rm{\Delta }}$ eq. (12) takes the simpler form ${\rm{\Omega }}^{\prime} g\approx {\rm{\Omega }}g^{\prime}$. Let us now verify the approximate formula (27) for the parameter regime corresponding to the confocal configuration with 1000 atoms. By setting *l* = 50 mm and *T*′_2_ = 800 ppm, we get $(g,g^{\prime} ,{\rm{\Delta }},{\rm{\Omega }},{\rm{\Omega }}^{\prime} ,\gamma ,\gamma ^{\prime} ,\gamma ^{\prime\prime} ,{\eta }_{{\rm{tot}}},\kappa )/(2\pi )=$ $(0.1,0.29,72.8,364,989,1.4,6.06,0.6,0.4,4.7\cdot {10}^{-3})$ MHz. These lead to (*ζ*_1_, *θ*_1_, *ζ*_2_, *θ*_2_)/(2*π*) = (1.3, 2.3, 1.2, 2.5) kHz and *δ*_2_/(2*π*) = 0.14 MHz. As mentioned earlier, the formula (27) is valid if the conditions ${\delta }_{2}\gg \kappa ,{\zeta }_{1},{\theta }_{1},{\zeta }_{2},{\theta }_{2}$ and ${\eta }_{{\rm{tot}}}\gg {\delta }_{2}$ are fulfilled. One can see that the first condition is fulfilled. However, the ratio *η*_tot_/*δ*_2_ is only 2.8. Nevertheless, the value of the figure of merit calculated using eq. (27), i.e., *F* = 0.95 is quite close to the value *F* = 0.92 obtained numerically using its definition.

So far, we have investigated the device working in the open mode. Let us now consider this device working in the closed mode. For the device working in the closed mode both lasers are turned off. The effective Hamiltonian derived with Ω = Ω′ = 0 is given by eq. (9). It is seen that there is no interaction between the *a* mode and the *b* mode, and therefore, photons do not leak out of the *a* mode through the *b* mode. The only destructive role played by atoms trapped inside the cavity is the increase of photon losses caused by the spontaneous emission from the atomic excited state |2〉. The decay of the *a* mode associated with the atomic spontaneous emission is described by an effective decay rate *κ*_*γ*_ ≈ *nγ*(*g*/Δ)^2^ [see eq. (8)]. We have found out that for the parameters values used above (*l* = 50 mm, *T*′_2_ = 800 ppm and *n* = 1000) this effective decay rate *κ*_*γ*_/(2*π*) = 2.9⋅10^−3^ MHz is less than the cavity decay rate associated with the absorption in the mirrors *κ*/(2*π*) = 4.7⋅10^−3^ MHz. Knowing *κ*_*γ*_, we can take atomic spontaneous emissions into account just by making the replacement *κ*→*κ*′ = *κ* + *κ*_*γ*_.

## Conclusion

We have studied a quantum system composed of a ⬦-type atom and an optical cavity supporting two electromagnetic field modes, in which this atom is permanently trapped. We have considered the case, where lower atomic transitions (see Fig. 1) are coupled to the field modes and upper atomic transitions are driven by classical laser fields. We have shown that this complex quantum system can be described by an effective Hamiltonian of the simple form given in eq. (3) if intensities of the lasers fields and the detuning are sufficiently large. In this case the ⬦-type atom intermediates in the interaction between these two modes of the cavity and creates a fully controllable effective coupling between these modes. One can control the effective coupling by lasers, which illuminate the atom. We have also shown that it is possible to obtain enhancement of the effective coupling of a factor $\sqrt{n}$ by trapping an ensemble of *n* ⬦-type atoms inside the cavity. We have presented two examples of applications of this system. The first application is a state transfer from one quantized mode to another. We have shown that the time of the state transfer is independent of the number of photons. Thus, it is possible to map a quantum state of one mode onto the other mode. As the second application of the system, we have presented a device which can be switched on demand to perform either as a low-Q cavity, or as a high-Q cavity. The ⬦-type atoms allow for fast switching between these two working modes just by switching the lasers on and off. Moreover, ⬦-type atoms make this device to be especially well suited for a bimodal cavity, which supports circularly polarised modes of the same or different polarisations and frequencies.

## Methods

### Reiter-Sørensen method

Initially, all atoms are prepared in the ground state. An atom can be found in one of the excited states, only if it absorbs a single photon. We want to achieve an effective coupling between field modes and no coupling between the modes and atoms. Therefore, the atomic excited states have to be populated only virtually. In this case, we can adiabatically eliminate the atomic excited states and use in calculations an effective Hamiltonian for the ground state subspace. To this end, we use the effective operator formalism for open quantum systems described in ref.^84^. Let us consider the single atom case first. The Hamiltonian describing a single atom can be easily obtained by simplifying eq. (1) and it reads

29 $$H={\rm{\Delta }}{\sigma }_{11}+{\rm{\Delta }}{\sigma }_{22}+2{\rm{\Delta }}{\sigma }_{33}+({\rm{\Omega }}{\sigma }_{23}+{\rm{\Omega }}^{\prime} {\sigma }_{13}+g{a}^{\dagger }{\sigma }_{02}+g^{\prime} {b}^{\dagger }{\sigma }_{01}+{\rm{h}}{\rm{.c}}{\rm{.}}).$$

The Lindblad operators representing spontaneous transitions from the atomic excited states are given by

30 $${L}_{1}=\sqrt{\gamma ^{\prime} }{\sigma }_{01},\,{L}_{2}=\sqrt{\gamma }{\sigma }_{02},\,{L}_{3}=\sqrt{{\gamma }_{3}}{\sigma }_{23},\,{L}_{4}=\sqrt{\gamma {^{\prime} }_{3}}{\sigma }_{13},$$

where *γ*, *γ*′, *γ*_3_ and *γ*′_3_ are spontaneous emission rates for the respective transitions. The master equation of Kossakowski-Lindblad form describing the evolution of this system is then given by

31 $$\dot{\rho }=-\,i[H,\rho ]+\sum _{j=1}^{4}[{L}_{j}\rho {L}_{j}^{\dagger }-\,\frac{1}{2}({L}_{j}^{\dagger }{L}_{j}\rho +\rho {L}_{j}^{\dagger }{L}_{j})].$$

The effective-operator formalism for open quantum systems^84^ reduces eq. (31) to an effective master equation, where the dynamics is restricted to the atomic ground state only. In order to apply the effective-operator formalism, we need to provide: the Lindblad operators, the Hamiltonian in the exited-state manifold *H*_e_, the ground-state Hamiltonian *H*_g_ (here *H*_g_ = 0.), and the perturbative (de-)excitations of the system *V*_+_ (*V*_−_). These are given by

32 $$\begin{array}{rcl}{H}_{{\rm{e}}} & = & {\rm{\Delta }}{\sigma }_{11}+{\rm{\Delta }}{\sigma }_{22}+2{\rm{\Delta }}{\sigma }_{33}+({\rm{\Omega }}{\sigma }_{23}+{\rm{\Omega }}^{\prime} {\sigma }_{13}+{\rm{h}}{\rm{.c}}{\rm{.}}),\\ {V}_{-} & = & g{a}^{\dagger }{\sigma }_{02}+g^{\prime} {b}^{\dagger }{\sigma }_{01},\\ {V}_{+} & = & ga{\sigma }_{20}+g^{\prime} b{\sigma }_{10}.\end{array}$$

The effective Hamiltonian and collapse operators can be derived using formulas^84^

33 $${H}_{{\rm{e}}{\rm{f}}{\rm{f}}}=\,-\,\frac{1}{2}{V}_{-}[{H}_{{\rm{N}}{\rm{H}}}^{-1}+{({H}_{{\rm{N}}{\rm{H}}}^{-1})}^{\dagger }]{V}_{+}+{H}_{{\rm{g}}},$$

34 $${L}_{{\rm{eff}}}^{(j)}={L}_{j}{H}_{{\rm{NH}}}^{-1}{V}_{+},$$

where

35 $${H}_{{\rm{NH}}}={H}_{{\rm{e}}}-\,\frac{i}{2}\sum _{j}{L}_{j}^{\dagger }{L}_{j}.$$

Assuming that all spontaneous emission rates are negligibly small compared with Ω, Ω′ and Δ we can approximate ${H}_{{\rm{NH}}}^{-1}$ by

36 $${H}_{{\rm{NH}}}^{-1}\approx {\alpha }_{1}{\sigma }_{11}+{\alpha }_{2}{\sigma }_{22}+{\alpha }_{3}({\sigma }_{12}+{\sigma }_{21})+{\alpha }_{4}{\sigma }_{33}+{\alpha }_{5}({\sigma }_{13}+{\sigma }_{31})+{\alpha }_{6}({\sigma }_{23}+{\sigma }_{32}),$$

where *α*_1_ = *ξ*(Ω^2^ − 2Δ^2^), *α*_2_ = *ξ*(Ω′^2^ − 2Δ^2^), *α*_3_ = −*ξ*ΩΩ′, *α*_4_ = −*ξ*Δ^2^, *α*_5_ = *ξ*ΔΩ′, *α*_6_ = *ξ*ΔΩ with *ξ* = 1/(Δ[Ω^2^ + Ω′^2^ − 2Δ^2^]). From the combined eqs (33) and (36) we derive the effective Hamiltonian

37 $${H}_{{\rm{eff}}}={\delta }_{0}({a}^{\dagger }a+{b}^{\dagger }b)+{\delta }_{1}{b}^{\dagger }b+{\delta }_{2}({a}^{\dagger }b+{b}^{\dagger }a),$$

where *δ*_0_ = −*g*^2^*α*_2_, *δ*_1_ = *g*^2^*α*_2_ − *g*′^2^*α*_1_ and *δ*_2_ = −*gg*′*α*_3_. By inserting eq. (36) into eq. (34) we obtain the effective Lindblad operators

38 $${L}_{{\rm{eff}}}^{\mathrm{(1)}}=\sqrt{\gamma ^{\prime} }[{\alpha }_{3}ga+{\alpha }_{1}g^{\prime} b],\,{L}_{{\rm{eff}}}^{\mathrm{(2)}}=\sqrt{\gamma }[{\alpha }_{2}ga+{\alpha }_{3}g^{\prime} b].$$

Unfortunately, deriving the expressions for operators ${L}_{{\rm{eff}}}^{\mathrm{(3)}}$ and ${L}_{{\rm{eff}}}^{\mathrm{(4)}}$ is more challenging than deriving ${L}_{{\rm{eff}}}^{\mathrm{(1)}}$ and ${L}_{{\rm{eff}}}^{\mathrm{(2)}}$. First of all, the action of the operators *L*_3_ and *L*_4_ takes the system state to one of the excited states |1〉 or |2〉, while all the excited states should be populated only virtually. In the single atom case after spontaneous emission from the excited state |3〉 it is necessary to reset the device, otherwise it will not work properly. Second, the effective operator formalism assumes that the excited states decay to the ground states only. We circumvent these obstacles by choosing such values of parameters that probabilities of occurrence of collapses described by *L*_3_ and *L*_4_ are negligibly small. We will give later conditions for the parameters, which allow us to neglect *L*_3_ and *L*_4_. Using this approximation, we can write the effective master equation as

39 $$\dot{\rho }=-\,i[{H}_{{\rm{eff}}},\rho ]+\sum _{j=1}^{2}\{{L}_{{\rm{eff}}}^{(j)}\rho {({L}_{{\rm{eff}}}^{(j)})}^{\dagger }-\,\frac{1}{2}[{L}_{{\rm{eff}}}^{(j)}{)}^{\dagger }{L}_{{\rm{eff}}}^{(j)}\rho +\rho {({L}_{{\rm{eff}}}^{(j)})}^{\dagger }{L}_{{\rm{eff}}}^{(j)}]\}.$$

### Alexanian-Bose method

Using the effective operator formalism and assuming that the upper level |3〉 can be neglected, we have obtained all needed formulas. Unfortunately we still do not know the limits in which these approximations are valid. In order to determine the limits we derive the effective Hamiltonian (3) using another method — a perturbative unitary transformation^85^. An incidental bonus is that this method provides new insights into the dynamics of the four-level atom in the diamond configuration. Let us start by decomposing the Hamiltonian (29) into two parts

40 $$H={H}_{0}+{H}_{1},$$

where

41 $${H}_{0}={\rm{\Delta }}{\sigma }_{11}+{\rm{\Delta }}{\sigma }_{22}+2{\rm{\Delta }}{\sigma }_{33}+({\rm{\Omega }}{\sigma }_{23}+{\rm{\Omega }}^{\prime} {\sigma }_{13}+{\rm{h}}{\rm{.c}}{\rm{.}}).$$

Diagonalizing *H*_0_ in the basis {|1〉, |2〉, |3〉} leads to the dressed states energies

42 $${\rm{\Delta }},\,(3{\rm{\Delta }}-{{\rm{\Omega }}}_{{\rm{R}}})\mathrm{/2},\,(3{\rm{\Delta }}+{{\rm{\Omega }}}_{{\rm{R}}})\mathrm{/2},$$

and the semiclassical dressed states

43 $$\begin{array}{rcl}|\mu \rangle  & = & {{\mathscr{N}}}_{\mu }(\,-\,{\rm{\Omega }}\mathrm{|1}\rangle +{\rm{\Omega }}^{\prime} \mathrm{|2}\rangle ),\\ |\varphi \rangle  & = & {{\mathscr{N}}}_{\varphi }(2{\rm{\Omega }}^{\prime} \mathrm{|1}\rangle +2{\rm{\Omega }}\mathrm{|2}\rangle +({\rm{\Delta }}-{{\rm{\Omega }}}_{{\rm{R}}})\mathrm{|3}\rangle ),\\ |\psi \rangle  & = & {{\mathscr{N}}}_{\psi }\mathrm{(2}{\rm{\Omega }}^{\prime} \mathrm{|1}\rangle +2{\rm{\Omega }}\mathrm{|2}\rangle +({\rm{\Delta }}+{{\rm{\Omega }}}_{{\rm{R}}}\mathrm{)|3}\rangle ),\end{array}$$

where Ω_R_ = (Δ^2^ + 4Ω^2^ + 4Ω′^2^)^1/2^, ${{\mathscr{N}}}_{\mu }$ = (Ω^2^ + Ω′^2^)^−1/2^, ${{\mathscr{N}}}_{\varphi }$ = (2Ω_R_(Ω_R_ − Δ))^−1/2^ and ${{\mathscr{N}}}_{\psi }$ = (2Ω_R_(Ω_R_ + Δ))^−1/2^. Now, using the new basis {|0〉, |*μ*〉, |*ϕ*〉, |*ψ*〉}, we express the Hamiltonian (29) as

44 $$\begin{array}{rcl}H & = & {\rm{\Delta }}{\sigma }_{\mu \mu }+(3{\rm{\Delta }}-{{\rm{\Omega }}}_{{\rm{R}}})\mathrm{/2}{\sigma }_{\varphi \varphi }+(3{\rm{\Delta }}+{{\rm{\Omega }}}_{{\rm{R}}})\mathrm{/2}{\sigma }_{\psi \psi }\\  &  & +\,({{\mathscr{N}}}_{\mu }g{\rm{\Omega }}^{\prime} {a}^{\dagger }{\sigma }_{0\mu }-2{{\mathscr{N}}}_{\varphi }g{\rm{\Omega }}{a}^{\dagger }{\sigma }_{0\varphi }+2{{\mathscr{N}}}_{\psi }g{\rm{\Omega }}{a}^{\dagger }{\sigma }_{0\psi }\\  &  & -\,{{\mathscr{N}}}_{\mu }g^{\prime} {\rm{\Omega }}{b}^{\dagger }{\sigma }_{0\mu }-2{{\mathscr{N}}}_{\varphi }g^{\prime} {\rm{\Omega }}^{\prime} {b}^{\dagger }{\sigma }_{0\varphi }+2{{\mathscr{N}}}_{\psi }g^{\prime} {\rm{\Omega }}^{\prime} {b}^{\dagger }{\sigma }_{0\psi }+{\rm{h}}{\rm{.c}}{\rm{.}}).\end{array}$$

Now we can eliminate atomic excited states |*μ*〉, |*ϕ*〉 and |*ψ*〉. To this end, we introduce a unitary transformation^85^

45 $$U=\exp (S),$$

where

46 $$\begin{array}{rcl}S & = & {\lambda }_{1}({\sigma }_{\mu 0}a-{a}^{\dagger }{\sigma }_{0\mu })+{\lambda }_{2}({\sigma }_{\varphi 0}a-{a}^{\dagger }{\sigma }_{0\varphi })\\  &  & +\,{\lambda }_{3}({\sigma }_{\psi 0}a-{a}^{\dagger }{\sigma }_{0\psi })+{\lambda }_{4}({b}^{\dagger }{\sigma }_{0\mu }-{\sigma }_{\mu 0}b)\\  &  & +\,{\lambda }_{5}({\sigma }_{\phi 0}b-{b}^{\dagger }{\sigma }_{0\varphi })+{\lambda }_{6}({\sigma }_{\psi 0}b-{b}^{\dagger }{\sigma }_{0\psi })\end{array}$$

and *λ*_*i*_ are dimensionless parameters such that ${\lambda }_{i}\sqrt{\langle {a}^{\dagger }a\rangle }$ and ${\lambda }_{i}\sqrt{\langle {b}^{\dagger }b\rangle }$ are very small compared to 1. These parameters will play the role of expansion parameters associated with respective excited states. For example, *λ*_1_ and *λ*_4_ are associated with the state |*μ*〉 (see eq. (46)).

We transform each operator in (44) using the Baker–Hausdorf lemma

47 $$X^{\prime} ={e}^{S}X{e}^{-S}=X+[S,X]+(1/\mathrm{2!})[S,[S,X]]+\ldots $$

If we choose *λ*_1_ = ${{\mathscr{N}}}_{\mu }$*g*Ω′/Δ, *λ*_2_ = $4{{\mathscr{N}}}_{\varphi }$*g*Ω/(3Δ − Ω_R_), *λ*_3_ = $4{{\mathscr{N}}}_{\psi }$*g*Ω/(3Δ + Ω_R_), *λ*_4_ = ${{\mathscr{N}}}_{\mu }$*g*′Ω/Δ, *λ*_5_ = $4{{\mathscr{N}}}_{\varphi }$*g*′Ω′/(3Δ − Ω_R_), *λ*_6_ = $4{{\mathscr{N}}}_{\psi }$*g*′Ω′/(3Δ + Ω_R_) then terms which are linear in the field operators vanish in the transformed Hamiltonian. If we moreover drop all terms much smaller than ${\lambda }_{1}^{2}{\rm{\Delta }}$ then we obtain the effective Hamiltonian (3). Note that both methods, *i.e*. Reiter-Sørensen method and Alexanian-Bose method, give exactly the same formula for the effective Hamiltonian, despite the fact that both are just approximations.

It is also worth to note that the parameters *λ*_*i*_ are given by the ratios of the effective coupling constants to the dressed state energies. The dressed state energies play the role of detunings in this dressed-state approach. So, ${\lambda }_{i}\ll 1$ means that the corresponding excited state is very far off resonance from the ground atomic state |0〉, and thus, its population is small. For instance, the smaller *λ*_1_ and *λ*_4_ are, the smaller is the population of the state |*μ*〉. Knowing this we can obtain the conditions (6).

### The multi-atom case

Let us now generalise the effective Hamiltonian to the case of *n* identical atoms trapped inside the cavity. For the sake of simplicity, we assume here that coupling strengths *g* and *g* ′ are the same for each atom in the ensemble. Note, however, that every atom in a Bose-Einstein condensate indeed experiences an identical coupling to the cavity mode^59^. The evolution of this multi-atom system is represented by the Hamiltonian

48 $$H=\sum _{k=1}^{n}\{{\rm{\Delta }}{\sigma }_{11}^{(k)}+{\rm{\Delta }}{\sigma }_{22}^{(k)}+2{\rm{\Delta }}{\sigma }_{33}^{(k)}+({\rm{\Omega }}{\sigma }_{23}^{(k)}+{\rm{\Omega }}^{\prime} {\sigma }_{13}^{(k)}+g{a}^{\dagger }{\sigma }_{02}^{(k)}+g^{\prime} {b}^{\dagger }{\sigma }_{01}^{(k)}+{\rm{h}}{\rm{.c}}{\rm{.}})\}.$$

Let us derive the effective Hamiltonian by using Alexanian-Bose method. To this end we rewrite the Hamiltonian (48) as

49 $$\begin{array}{rcl}H & = & \sum _{k=1}^{n}\{{\rm{\Delta }}{\sigma }_{\mu \mu }^{(k)}+\mathrm{(3}{\rm{\Delta }}-{{\rm{\Omega }}}_{{\rm{R}}}\mathrm{)/2}{\sigma }_{\varphi \varphi }^{(k)}+\mathrm{(3}{\rm{\Delta }}+{{\rm{\Omega }}}_{{\rm{R}}}\mathrm{)/2}{\sigma }_{\psi \psi }^{(k)}\\  &  & +\,({{\mathscr{N}}}_{\mu }g{\rm{\Omega }}^{\prime} {a}^{\dagger }{\sigma }_{0\mu }^{(k)}-2{{\mathscr{N}}}_{\varphi }g{\rm{\Omega }}{a}^{\dagger }{\sigma }_{0\varphi }^{(k)}+2{{\mathscr{N}}}_{\psi }g{\rm{\Omega }}{a}^{\dagger }{\sigma }_{0\psi }^{(k)}\\  &  & -\,{{\mathscr{N}}}_{\mu }g^{\prime} {\rm{\Omega }}{b}^{\dagger }{\sigma }_{0\mu }^{(k)}-2{{\mathscr{N}}}_{\varphi }g^{\prime} {\rm{\Omega }}^{\prime} {b}^{\dagger }{\sigma }_{0\varphi }^{(k)}+2{{\mathscr{N}}}_{\psi }g^{\prime} {\rm{\Omega }}^{\prime} {b}^{\dagger }{\sigma }_{0\psi }^{(k)}+{\rm{h}}{\rm{.c}}{\rm{.}})\}\end{array}$$

and introduce a unitary transformation *U* = exp(*S*), where

50 $$\begin{array}{rcl}S & = & \sum _{k=1}^{n}\{{\lambda }_{1}({\sigma }_{\mu 0}^{(k)}a-{a}^{\dagger }{\sigma }_{0\mu }^{(k)})+{\lambda }_{2}({\sigma }_{\varphi 0}^{(k)}a-{a}^{\dagger }{\sigma }_{0\varphi }^{(k)})\\  &  & +{\lambda }_{3}({\sigma }_{\psi 0}^{(k)}a-{a}^{\dagger }{\sigma }_{0\psi }^{(k)})+{\lambda }_{4}({b}^{\dagger }{\sigma }_{0\mu }^{(k)}-{\sigma }_{\mu 0}^{(k)}b)\\  &  & +{\lambda }_{5}({\sigma }_{\varphi 0}^{(k)}b-{b}^{\dagger }{\sigma }_{0\varphi }^{(k)})+{\lambda }_{6}({\sigma }_{\psi 0}^{(k)}b-{b}^{\dagger }{\sigma }_{0\psi }^{(k)})\}.\end{array}$$

If we set *λ*_1_ = ${{\mathscr{N}}}_{\mu }$*g*Ω′/Δ, *λ*_2_ = $4{{\mathscr{N}}}_{\varphi }$*g*Ω/(3Δ − Ω_R_), *λ*_3_ = $4{{\mathscr{N}}}_{\psi }$*g*Ω/(3Δ + Ω_R_), *λ*_4_ = ${{\mathscr{N}}}_{\mu }$*g*′Ω/Δ, *λ*_5_ = $4{{\mathscr{N}}}_{\varphi }$*g*′Ω′/(3Δ − Ω_R_), *λ*_6_ = $4{{\mathscr{N}}}_{\psi }$*g*′Ω′/(3Δ + Ω_R_), assume that the conditions (6) are satisfied and drop all terms much smaller than ${\lambda }_{1}^{2}{\rm{\Delta }}$ then the transformed Hamiltonian takes the form

51 $$H^{\prime} ={H}_{{\rm{eff}}}\,\sum _{k=1}^{n}{\sigma }_{00}^{(k)}+{\beta }_{1}(\sum _{k=1}^{n}{\sigma }_{\mu 0}^{(k)})(\sum _{k=1}^{n}{\sigma }_{0\mu }^{(k)})+{\beta }_{2}\{(\sum _{k=1}^{n}{\sigma }_{\mu 0}^{(k)})[\sum _{k=1}^{n}{\sigma }_{0\varphi }^{(k)}+\sum _{k=1}^{n}{\sigma }_{0\psi }^{(k)}]+{\rm{h}}{\rm{.c}}{\rm{.}}\},$$

where *H*_eff_ is the effective Hamiltonian (37) describing the evolution of the system in the single-atom case, *β*_1_ = *g*^2^*g*′^2^/(Δ(*g*^2^ + *g*′^2^)) and ${\beta }_{2}=gg^{\prime} ({g}^{2}-g{^{\prime} }^{2})/(\sqrt{2}{\rm{\Delta }}({g}^{2}+g{^{\prime} }^{2}))$. Initially all atoms are prepared in the ground state. It is seen that there is no operator in the Hamiltonian (51), which can change this atomic state. Hence all atoms remain in their ground state during the evolution. Therefore we can drop all terms describing excitation exchange between different atoms, i.e., terms containing operators ${\sigma }_{m0}^{(i)}{\sigma }_{0l}^{(j)}$, where *m*, *l* = *μ*, *ψ*, *ϕ*. Of course, we can also drop all terms containing operators ${\sigma }_{\mu \mu }^{(k)}$. Then we obtain

52 $$H^{\prime} =n{H}_{{\rm{eff}}}{\Pi }_{k=1}^{n}{\sigma }_{00}^{(k)},$$

So, in this more general case the effective Hamiltonian is still given by eq. (37), but with

53 $${\delta }_{0}=-\,n{g}^{2}{\alpha }_{2},\,{\delta }_{2}=-\,ngg^{\prime} {\alpha }_{3},\,{\rm{and}}\,{\delta }_{1}=n({g}^{2}{\alpha }_{2}-g{^{\prime} }^{2}{\alpha }_{1}).$$

Let us also derive the effective Hamiltonian using the Holstein-Primakoff transformation^86^—a standard method in the study of multi-atom systems. We start from the Hamiltonian (49). As mentioned earlier, only two atomic levels, *i.e*. |0〉 and |*μ*〉, are enough to describe the evolution of the system in the limit of high-intensity classical fields (see Results). Therefore we assume that the conditions (6) are satisfied and we approximate the Hamiltonian (49) by

54 $$H=\frac{{\rm{\Delta }}}{2}\sum _{k=1}^{n}{\sigma }_{3}^{(k)}+({{\mathscr{N}}}_{\mu }g{\rm{\Omega }}^{\prime} {a}^{\dagger }\sum _{k=1}^{n}{\sigma }_{0\mu }^{(k)}-{{\mathscr{N}}}_{\mu }g^{\prime} {\rm{\Omega }}{b}^{\dagger }\sum _{k=1}^{n}{\sigma }_{0\mu }^{(k)}+{\rm{h}}{\rm{.c}}{\rm{.}}),$$

where ${\sigma }_{3}^{(k)}={\sigma }_{\mu \mu }^{(k)}-{\sigma }_{00}^{(k)}$. Next, we introduce the operators ${S}_{z}={2}^{-1}{\sum }_{k=1}^{n}{\sigma }_{3}^{(k)}$, ${S}_{-}={\sum }_{k=1}^{n}{\sigma }_{0\mu }^{(k)}$, ${S}_{+}={\sum }_{k=1}^{n}{\sigma }_{\mu 0}^{(k)}$ and rewrite the Hamiltonian as

55 $$H={\rm{\Delta }}{S}_{z}+({{\mathscr{N}}}_{\mu }g{\rm{\Omega }}^{\prime} {a}^{\dagger }{S}_{-}-{{\mathscr{N}}}_{\mu }g^{\prime} {\rm{\Omega }}{b}^{\dagger }{S}_{-}+{\rm{h}}{\rm{.c}}{\rm{.}}).$$

Now we can use the Holstein-Primakoff approximation defined by

56 $${S}_{z}=\frac{n}{2}-\,{c}^{\dagger }c,\,{S}_{-}=\sqrt{n}{(1-{c}^{\dagger }c/n)}^{\mathrm{1/2}}c\approx \sqrt{n}c,\,{\rm{and}}\,{S}_{+}=\sqrt{n}{c}^{\dagger }{(1-{c}^{\dagger }c/n)}^{\mathrm{1/2}}\approx \sqrt{n}{c}^{\dagger }.$$

The Hamiltonian can be written in terms of a bosonic operator *c* as

57 $$H=-\,{\rm{\Delta }}{c}^{\dagger }c+({{\mathscr{N}}}_{\mu }g{\rm{\Omega }}^{\prime} \sqrt{n}{a}^{\dagger }c-{{\mathscr{N}}}_{\mu }g^{\prime} {\rm{\Omega }}\sqrt{n}{b}^{\dagger }c+{\rm{h}}{\rm{.c}}{\rm{.}}).$$

By assuming that the conditions (6) are satisfied we also assume that the average number of bosonic excitations $\langle {c}^{\dagger }c\rangle$ is small. Thus we can adiabatically eliminate the bosonic operator *c*. To this end, we derive formula for *c* from $\dot{c}=i[H,c]=0$^87^. In this way we obtain

58 $$c=({{\mathscr{N}}}_{\mu }g{\rm{\Omega }}^{\prime} \sqrt{n}a-{{\mathscr{N}}}_{\mu }g^{\prime} {\rm{\Omega }}\sqrt{n}b)/{\rm{\Delta }}.$$

Substituting eq. (58) into the Hamiltonian (57) results in

59 $${\tilde{H}}_{{\rm{eff}}}={\tilde{\delta }}_{0}({a}^{\dagger }a+{b}^{\dagger }b)+{\tilde{\delta }}_{1}{b}^{\dagger }b+{\tilde{\delta }}_{2}({a}^{\dagger }b+{b}^{\dagger }a),$$

where $${\tilde{\delta }}_{0}=n{g}^{2}{\rm{\Omega }}{^{\prime} }^{2}\tilde{\xi }$$, $${\tilde{\delta }}_{1}=n(g{^{\prime} }^{2}{{\rm{\Omega }}}^{2}-{g}^{2}{\rm{\Omega }}{^{\prime} }^{2})\tilde{\xi }$$, $${\tilde{\delta }}_{2}=-\,ngg^{\prime} {\rm{\Omega }}{\rm{\Omega }}^{\prime} \tilde{\xi }$$ and $$\tilde{\xi }=1/({\rm{\Delta }}({{\rm{\Omega }}}^{2}+{\rm{\Omega }}{^{\prime} }^{2}))$$.

**Figure 7:**
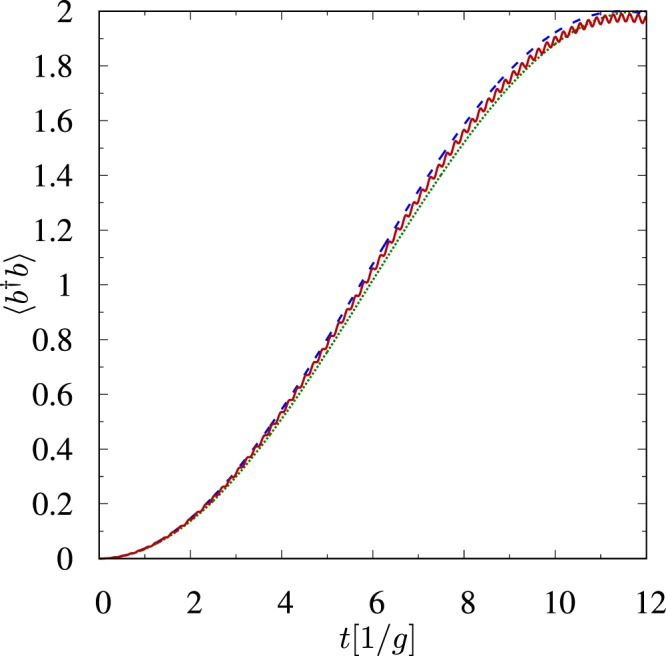
The average photon number in the *b* mode calculated numerically using the general Hamiltonian (48) (solid curve), the effective Hamiltonian (3) (dashed curve) and the effective Hamiltonian (59) (dotted curve). The parameters regime is (*g*′, Δ, Ω, Ω′)/*g* = (1, 34, 180, 180), where *g*/2*π* = 10 MHz and the number of atoms is set to 9.

The effective Hamiltonian (59) is in agreement with the effective Hamiltonian (3) in the limit of high-intensity classical fields. In order to show this agreement we have calculated numerically the average photon number in the mode represented by the annihilation operator *b* for an ensemble of 9 atoms using both these effective Hamiltonians and the general Hamiltonian (48). We have assumed that initially the *b* mode is in a vacuum state, while the *a* mode is prepared in the state |2〉_*A*_ and all atoms are prepared in their ground state. The values of the parameters are chosen such that the conditions (6) are satisfied. It is seen from Fig. 7 that the general Hamiltonian (48) describing the multi-atom case is well approximated by both effective Hamiltonians.

### Interaction with an external field

The two cavity modes interact according to the effective Hamiltonian (3), which in a frame rotating at *δ*_0_ takes the form ${H}_{{\rm{eff}}}={\delta }_{1}{b}^{\dagger }b+{\delta }_{2}({a}^{\dagger }b+{b}^{\dagger }a)$. The photon emission from the mode, represented by *b*, into the waveguide is described by the Lindblad operator ${L}_{\eta }=\sqrt{\eta }b$. The absorption in the mirrors for this mode is modelled as the photon emission into an inaccessible mode and described by ${L}_{\eta ^{\prime} }=\sqrt{\eta ^{\prime} }b$. The losses in the mirrors for the *a* mode are taken into account in the same manner. The photon absorption from the *a* mode is described by the operator ${L}_{\kappa }=\sqrt{\kappa }a$. Although the simplified model does not include atomic variables, spontaneous emissions from the excited atomic states |1〉 and |2〉 are taken into account by assuming that there are two inaccessible travelling modes, into which photons from both modes can be emitted in the way described by the Lindblad operators ${L}_{{\rm{eff}}}^{\mathrm{(1)}}$ and ${L}_{{\rm{eff}}}^{\mathrm{(2)}}$. The device working in the open mode has to transfer the state of the *a* mode to the waveguide. In order to calculate a quantity, which measures how close the output field into waveguide is to the initial *a* mode field, it is necessary to describe the interaction of the quantum system with the accessible travelling mode. To this end, we use the input-output theory^88–90^, because it is perfectly suitable for the scheme illustrated in Fig. 4. We have followed the treatment of ref.^89^ to derive the Heisenberg-Langevin equations for this scheme. These equations take the form

60 $$\begin{array}{rcl}\dot{a} & = & (\,-\,i{\delta }_{2}+(\sqrt{{\zeta }_{1}{\theta }_{1}}+\sqrt{{\zeta }_{2}{\theta }_{2}}\mathrm{)/2)}b+(\kappa +{\zeta }_{1}+{\zeta }_{2})a\mathrm{/2}\\  &  & -\,\sqrt{\kappa }{a}_{\kappa }-\sqrt{{\zeta }_{1}}c-\sqrt{{\zeta }_{2}}d\,,\\ \dot{b} & = & (\,-\,i{\delta }_{2}+(\sqrt{{\zeta }_{1}{\theta }_{1}}+\sqrt{{\zeta }_{2}{\theta }_{2}}\mathrm{)/2)}a+(\,-\,i{\delta }_{1}+({\eta }_{{\rm{tot}}}+{\theta }_{1}+{\theta }_{2})\mathrm{/2})b\\  &  & -\,\sqrt{\eta }{b}_{\eta }-\sqrt{\eta ^{\prime} }{b}_{\eta ^{\prime} }-\sqrt{{\theta }_{1}}c-\sqrt{{\theta }_{2}}d,\end{array}$$

where *a*_*κ*_(*t*), *b*_*η*_′(*t*), *c*(*t*) and *d*(*t*) are output field operators of inaccessible travelling modes, *b*_*η*_(*t*) is the output field operator of the waveguide mode and ${\zeta }_{1}=n\gamma ^{\prime} {\alpha }_{3}^{2}{g}^{2}$, ${\theta }_{1}=n\gamma ^{\prime} {\alpha }_{1}^{2}g{^{\prime} }^{2}$, ${\zeta }_{2}=n\gamma {\alpha }_{2}^{2}{g}^{2}$, ${\theta }_{2}=n\gamma {\alpha }_{3}^{2}g{^{\prime} }^{2}$, *η*_tot_ = *η*′ + *η*. The matrix form of eq. (60) is given by

61 $$\dot{{v}}={Mv}-{{v}}_{{\rm{out}}},$$

with

62 $${M}\equiv [\begin{array}{cc}\frac{\kappa +{\zeta }_{1}+{\zeta }_{2}}{2} & \frac{\sqrt{{\zeta }_{1}{\theta }_{1}}+\sqrt{{\zeta }_{2}{\theta }_{2}}}{2}-\,i{\delta }_{2}\\ \frac{\sqrt{{\zeta }_{1}{\theta }_{1}}+\sqrt{{\zeta }_{2}{\theta }_{2}}}{2}-\,i{\delta }_{2} & \frac{{\eta }_{{\rm{tot}}}+{\theta }_{1}+{\theta }_{2}}{2}-\,i{\delta }_{1}\end{array}],$$

where *v* = [*a*, *b*]^T^ and $${{v}}_{{\rm{out}}}={[\sqrt{\kappa }{a}_{\kappa }+\sqrt{{\zeta }_{1}}c+\sqrt{{\zeta }_{2}}d,\sqrt{\eta }{b}_{\eta }+\sqrt{\eta ^{\prime} }{b}_{\eta ^{\prime} }+\sqrt{{\theta }_{1}}c+\sqrt{{\theta }_{2}}d]}^{{\rm{T}}}$$.

### Figure of merit

Now, we can follow closely the treatment of Tufarelli *et al*.^59^ to get the figure of merit of the scheme. First, we have to define the bosonic operator for the waveguide field travelling away from the device

63 $${f}_{{\rm{out}}}\equiv {\int }_{0}^{\infty }u(\tau ){b}_{\eta }(\tau )d\tau ,$$

with *u*(*τ*) being a temporal profile of the form

64 $$u(\tau )\equiv \frac{{[{e}^{-{M}\tau }]}_{1,2}}{\sqrt{{\int }_{0}^{\infty }|[{e}^{-{M}\tau }{]}_{1,2}{|}^{2}d\tau }}.$$

Next, we introduce the bosonic operator *h*_ext_ representing all inaccessible travelling modes. We do not need to know the specific form of *h*_ext_ in our calculations. Then we can relate the annihilation operator *a* at the time *t* = 0 to the output modes using the formula

65 $$a(0)=\sqrt{F}{f}_{{\rm{out}}}-\sqrt{1-F}{h}_{{\rm{ext}}},$$

where

66 $$F=\eta {\int }_{0}^{\infty }|{[{e}^{-{M}\tau }]}_{1,2}{|}^{2}d\tau .$$

It is worth to note the similarity between eq. (65) and a unitary transformation representing a beam splitter of transmittance *F*. This similarity allows us to consider an abstract beam splitter described by relations

67 $$a(0)=\sqrt{F}{f}_{{\rm{out}}}-\sqrt{1-F}{h}_{{\rm{ext}}},$$

68 $${a}_{{\rm{vac}}}(0)=\sqrt{1-F}{f}_{{\rm{out}}}+\sqrt{F}{h}_{{\rm{ext}}}.$$

The abstract mode *a*_vac_(0) must be empty, because the total excitation number has to be conserved, *i.e*., the initial number of photons inside the *a* mode has to be equal to the total number of photons inside outgoing modes *f*_out_ and *h*_ext_. Using the abstract beam-splitter model of the device it is easy to get formula for *f*_out_:

69 $${f}_{{\rm{out}}}=\sqrt{F}a(0)+\sqrt{1-F}{a}_{{\rm{vac}}}(0).$$

The parameter *F* satisfies $0\le F\le 1$ and, as it is easy to see from eq. (69), it can work as a figure of merit, because as *F* gets closer to one, the output field *f*_out_ gets closer to the initial field *a*(0). This fact is especially clearly seen in the Schrödinger picture^59^

70 $${\rho }_{{\rm{out}}}={e}^{(1-F) {\mathcal L} }{\rho }_{0},$$

where *ρ*_0_ is the initial state of the *a* mode, *ρ*_out_ is the final state of the *f*_out_ mode and the Liouvillian is given by

71 $$ {\mathcal L} \rho =\frac{1}{2}(2a\rho {a}^{\dagger }-{a}^{\dagger }a\rho -\rho {a}^{\dagger }a).$$

In order to investigate how well the initial quantum state can be extracted from the cavity using the device presented in Fig. 4, we have to express the figure of merit *F* as a function of parameters of this device. It can be done using the method presented in ref.^59^. First, we express the figure of merit as

72 $$F=\eta {X}_{1,2,2,1}({M}),$$

where

73 $${{\mathscr{X}}}_{1,2,2,1}({M})={\int }_{0}^{\infty }{[{e}^{-{M}\tau }]}_{1,2}{[{e}^{-{{M}}^{\dagger }\tau }]}_{2,1}d\tau $$

is an element of the tensor ${\mathscr{X}}$. We can express this tensor in the matrix form as

74 $$X({M})={\int }_{0}^{\infty }{e}^{-{M}\tau }\otimes {e}^{-{{M}}^{\dagger }\tau }d\tau ,$$

where ⊗ indicates the Kronecker product. Since ${\mathscr{X}}({M})$ is the solution to a Sylvester equation, we can obtain all elements of ${\mathscr{X}}({M})$ just by solving linear system of equations

75 $$({M}\otimes I)\,{\mathscr{X}}({M})+{\mathscr{X}}({M})(I\otimes {{M}}^{\dagger })=I\otimes I,$$

where *I* indicates the 2 × 2 identity matrix. In this way we derive the formula for ${{\mathscr{X}}}_{\mathrm{1,2,2,1}}({M})$, which we insert into eq. (72). Unfortunately, the obtained expression is too complex to be useful, and thus, it is necessary to resort to further approximations. If we assume that ${\eta }_{{\rm{tot}}}\gg {\delta }_{2}\gg \kappa ,{\zeta }_{1},{\theta }_{1},{\zeta }_{2},{\theta }_{2}$ then the figure of merit can be well approximated by

76 $$F=\frac{\eta }{{\eta }_{{\rm{tot}}}}[1-\,\frac{{(\sqrt{{\zeta }_{1}{\theta }_{1}}+\sqrt{{\zeta }_{2}{\theta }_{2}})}^{2}}{2{\delta }_{2}^{2}}-\frac{{\eta }_{{\rm{tot}}}(\kappa +{\zeta }_{1}+{\zeta }_{2})}{4{\delta }_{2}^{2}+{\eta }_{{\rm{tot}}}(\kappa +{\zeta }_{1}+{\zeta }_{2})}].$$
